# Transforming Growth Factor β Signaling Overcomes Dasatinib Resistance in Lung Cancer

**DOI:** 10.1371/journal.pone.0114131

**Published:** 2014-12-11

**Authors:** Edna Gordian, Jiannong Li, Yuri Pevzner, Melanie Mediavilla-Varela, Kimberly Luddy, Kim Ohaegbulam, Kenyon G. Daniel, Eric B. Haura, Teresita Muñoz-Antonia

**Affiliations:** 1 Molecular Oncology Program, H. Lee Moffitt Cancer Center & Research Institute, Tampa, Florida 33612, United States of America; 2 Department of Thoracic Oncology, H. Lee Moffitt Cancer Center & Research Institute, Tampa, Florida 33612, United States of America; 3 Chemical Biology Core, H. Lee Moffitt Cancer Center & Research Institute, Tampa, Florida 33612, United States of America; 4 Immunology Program, H. Lee Moffitt Cancer Center & Research Institute, Tampa, Florida 33612, United States of America; 5 Cancer Imaging & Metabolism Program, H. Lee Moffitt Cancer Center & Research Institute, Tampa, Florida 33612, United States of America; 6 Department of Microbiology and Immunology, Albert Einstein College of Medicine, Bronx, New York 10461, United States of America; Wayne State University, United States of America

## Abstract

Lung cancer is the second most common cancer and the leading cause of cancer-related deaths. Despite recent advances in the development of targeted therapies, patients with advanced disease remain incurable, mostly because metastatic non-small cell lung carcinomas (NSCLC) eventually become resistant to tyrosine kinase inhibitors (TKIs). Kinase inhibitors have the potential for target promiscuity because the kinase super family is the largest family of druggable genes that binds to a common substrate (ATP). As a result, TKIs often developed for a specific purpose have been found to act on other targets. Drug affinity chromatography has been used to show that dasatinib interacts with the TGFβ type I receptor (TβR-I), a serine-threonine kinase. To determine the potential biological relevance of this association, we studied the combined effects of dasatinib and TGFβ on lung cancer cell lines. We found that dasatinib treatment alone had very little effect; however, when NSCLC cell lines were treated with a combination of TGFβ and dasatinib, apoptosis was induced. Combined TGFβ-1 + dasatinib treatment had no effect on the activity of Smad2 or other non-canonical TGFβ intracellular mediators. Interestingly, combined TGFβ and dasatinib treatment resulted in a transient increase in p-Smad3 (seen after 3 hours). In addition, when NSCLC cells were treated with this combination, the pro-apoptotic protein BIM was up-regulated. Knockdown of the expression of Smad3 using Smad3 siRNA also resulted in a decrease in BIM protein, suggesting that TGFβ-1 + dasatinib-induced apoptosis is mediated by Smad3 regulation of BIM. Dasatinib is only effective in killing EGFR mutant cells, which is shown in only 10% of NSCLCs. Therefore, the observation that wild-type EGFR lung cancers can be manipulated to render them sensitive to killing by dasatinib could have important implications for devising innovative and potentially more efficacious treatment strategies for this disease.

## Introduction

Lung cancer is the second most common cancer and accounts for about 15% of all cancer diagnoses. Despite recent advances in the development of targeted therapies, patients with advanced disease remain incurable. Due to the genetic diversity within tumors, cells with activated alternative growth pathways eventually emerge; thus understanding the mechanisms by which different pathways are switched on and off is important in devising novel targeted therapies. Although some cancers are initially very sensitive to tyrosine kinase inhibitors (TKIs), resistance eventually develops. For example, a majority of metastatic non-small cell lung cancer (NSCLC) patients with EGFR-activating mutations respond to treatment with erlotinib; however, all patients ultimately progress. Therefore, alternate therapies are urgently needed for patients with EGFR mutations who initially respond to EGFR TKIs therapies but eventually develop resistance, as well as for patients who exhibit the wild-type EGFR genotype [1]. This resistance could be partly because of the complexity that characterizes the signaling of these types of proteins as well as the heterogeneity of lung adenocarcinomas [2]. Dasatinib, a TKI with multiple kinase targets, is currently being tested to treat different malignancies where these targets are overexpressed, including chronic myelogenous leukemia and breast and lung cancers [3], [4], [5]. Clinical trials have tested the efficacy of dasatinib in NSCLC as a single agent [6], in combination with currently used chemotherapy regimens such as the epidermal growth factor receptor (EGFR) inhibitor erlotinib [7], and in patients who have developed resistance to erlotinib and gefitinib [8]. Song *et al* demonstrated that dasatinib induces apoptosis in a number of NSCLC cells that exhibit a mutant EGFR phenotype; however, this effect was not observed in NSCLC cell lines with a wild-type EGFR phenotype [9].

Transforming growth factor β (TGFβ) is a cytokine involved in numerous cellular processes, including growth, proliferation, adhesion, migration, and apoptosis. In addition, loss of responsiveness to TGFβ-1 has been correlated with tumorigenicity in many different cancer types [10]. TGFβ signal transduction begins with ligand binding to the TGFβ type II receptor (TβR-II) followed by recruitment of the type I receptor (TβR-I) and formation of a hetero-oligomeric complex of TGFβ-1, TβR-II, and TβR-I [11]. After complex formation, the constitutively autophosphorylated TβR-II phosphorylates TβR-I, initiating a phosphorylation cascade of downstream cytoplasmic substrates, including the Smad proteins, with subsequent activation of target genes [10]. The cross talk between the TGFβ pathway and many other signal transduction pathways results in modification of the original TGFβ signal through non-canonical pathways and is used to explain the multiple effects of TGFβ [12], [13], [14]. In normal epithelial cells, TGFβ inhibits cell proliferation and induces apoptosis, thereby acting as a tumor suppressor; however, in many cancer types, TGFβ acts as a tumor promoter (cell invasion, metastasis, immune regulation, and microenvironment modification)[15].

Drug affinity chromatography experiments revealed that dasatinib, originally developed as a SRC inhibitor [16], interacts with over 40 kinases, including tyrosine kinases, receptor tyrosine kinases, serine/threonine kinases, and MAP kinases. One of the serine/threonine kinases that was identified using this approach was the TGFβ type I receptor (TβR-I) [17]. In order to determine the potential biological relevance of this association, we studied the combined effects of dasatinib and TGFβ on NSCLC cell lines. We found that dasatinib treatment alone had very little effect; however, when NSCLC cell lines were treated with a combination of TGFβ and dasatinib, apoptosis was induced. Dasatinib is only effective in killing EGFR mutant cells [9], which accounts for 10% of NSCLCs; therefore, the observation that lung cancers can be manipulated to render them sensitive to killing by dasatinib could have important implications for devising innovative and potentially more efficacious treatment strategies for this disease.

## Materials and Methods

### Cell culture

The human lung adenocarcinoma NCI H292 and A549 cell lines were obtained from the American Type Culture Collection (Manassas, VA) and cultured in RPMI-1640 medium (Thermo Scientific, Waltham, MA) supplemented with 10% fetal bovine serum (Atlanta Biologicals, Inc, Lawrenceville, GA), 100 U/mL penicillin, 100 µg/mL streptomycin, and 1 mM glutamine. The cell lines were maintained in a humid incubator at 37°C and 5% CO_2_.

### Antibodies and compounds

Polyclonal anti-Shc1 antibody was obtained from Thermo Scientific Pierce (Rockford, IL), and polyclonal anti-MADH7 (Smad7) antibody was purchased from Abcam Inc. (Cambridge, MA). Anti-HDAC2 was purchased from Millipore (Billerica, MA). The following antibodies were purchased from Cell Signaling Technology (Danvers, MA): anti-pSma2 (linker and COOH specific phosphorylation), anti-Smad2, anti-pSmad3, anti-Smad3, anti-pAKT, anti-pERK, anti-BIM, anti-PARP, and anti-GAPDH. Recombinant human TGFβ-1 protein was purchased from R&D Systems (Minneapolis, MN) and reconstituted in 4 mM HCL and 1 mg/mL bovine serum albumin solution. Dasatinib and erlotinib were obtained from ChemieTek (Indianapolis, IN) and diluted in DMSO. LY-364947 was purchase from Sigma-Aldrich (St. Louis, MO). AZD0530 was obtained from AstraZeneca (London, UK).

### TβR-I docking studies

The crystal structure of TβR-1 in complex with FKBP12 peptide and dorsomorphin (PDB ID: 3H9R) was used for starting coordinates [18]. All atoms not belonging to the protein were manually deleted using Schrodinger's Maestro [19] molecular modeling environment. Protein was prepared using Schrodinger's protein preparation protocol [20]. The initial stage of protein preparation included addition of the hydrogen atoms assignment of proper bond orders to all atoms, followed by creation of disulfide bonds, addition of missing side chains using Prime [21] software, and deletion of all water molecules. After the initial preparation, the protein structure was optimized to reflect a pH of 7.0 using PROPKA [22], [23] software followed by minimization of the entire structure until heavy atoms converged to root-mean-square deviation of 0.30 Å.

With the use of Desmond [24], [25] software, the prepared protein structure was solvated in a bath containing TIP3P [26] water molecules and chloride ions in a cubic box, such that there was a solvent buffer of 10 Å between the protein surface and the edge of the solvent box. A molecular dynamics simulation was then performed on the system. Using a Desmond's default relaxation and heating protocol, we heated the system to 310 K and equilibrated for a total chemical time of 10 ns using constant pressure and temperature ensemble. Interatomic coulombic interactions were calculated for atom pairs separated by up to 13 Å, while smooth particle mesh Ewald was used to account for interactions beyond the cutoff. Trajectory analysis indicated that the system reached equilibrium at around 4.5 ns of chemical time. Nine snapshots of the system corresponding to 4.613 ns, 6.005 ns, 6.394 ns, 7.123 ns, 7.550 ns, 8.050 ns, 8.789 ns, 9.538 ns, and 10.00 ns were picked at random. Using Maestro's average structure script, the snapshot representing the system at 7.123 ns was picked as the representative structure and used in the subsequent docking studies.

The ligands bosutinib, dasatinib, dorsomorphin, and LY-364947 were processed with LigPrep [27]. The processing ensured proper bond orders and, using the Epik [28], [29] module, generated all possible tautomers of the ligands to reflect a pH in the range of 5.0 to 9.0. SiteMap [30], [31] software was used to probe the surface of the TβR-1 for possible binding pockets. A total of five binding pockets were identified; these were ranked based on characteristics that included hydrophobicity, solvent exposure, and hydrogen bonding potential. Initial Glide [32], [33] SP (standard precision) followed by XP (extra precision) docking of the four compounds was performed to the top 3 binding sites. Based on this initial docking, Site 1 was chosen as the more likely binding site. Site 1, which correlates to the pocket where dorsomorphin was described, was also ranked highest by SiteMap.

Subsequent docking studies were carried out using Induced Fit Docking (IFD) [32], [34] workflow in Maestro. IFD workflow accounts for the receptor flexibility by sampling orientations of the site chains within the binding region upon docking of the ligand. Multiple ligand conformations are docked in such manner and scored using Glide. Each ligand was docked into Site 1 defined by the ligand's best scoring pose from the initial SP docking into Site 1. For each ligand IFD scored and reported multiple (∼50) protein-ligand conformations from which the average IFD score was calculated.

### Proliferation/Cytotoxiticy

A549 cells were seeded in 96-well plates at 5×10^3^ cells per well in triplicate. After 24 hours, cells were treated with the indicated concentrations of TGFβ-1, dasatinib, or TGFβ + dasatinib combined. After a 48-hour incubation, proliferation/cytotoxicity was measured by adding CellTiter 96 One Solution (Promega Corporation, Madison, WI). After addition, cells were left to incubate for 1 hour, and absorbance was measured at 490 nm in a BioTeK EL808 microplate reader (BioTek Instruments Inc, Winooski, VT). Treated samples were normalized to untreated controls, with results shown as percentages.

### Western blotting

Whole-cell protein extraction was performed by scrapping the cells in cold 1X phosphate-buffered saline (PBS), followed by sonication and lysis in 1X CHAPS buffer (Cell Signaling Technology). To determine the subcellular localization of Smads proteins, cells were fractionated into nuclear and cytoplasmic components as previously described [35]. Protein concentrations were determined using the Bradford Assay (Bio-Rad, Hercules, CA). Protein lysates were resolved by sodium dodecyl sulfate–polyacrylamide gel electrophoresis (SDS-PAGE), transferred to polyvinylidene fluoride membrane (Millipore Corporation, Billerica, MA), blocked for 1 hour in 1X TBST containing 5% nonfat milk, and incubated overnight in corresponding primary antibody at 4°C. Blots were finished by incubation with horseradish peroxidase-labeled secondary antibody and developed using Amersham ECL Prime Western Blotting Detection Reagent (GE Healthcare Life Sciences, Pittsburg, PA).

### Caspase assay

A549 cells were seeded in 96-well plates at 5×10^3^ cells per well. After 24 hours, cells were treated with the indicated concentrations of TGFβ-1, dasatinib, or TGFβ + dasatinib combined, and Cell Player 96-well kinetic caspase 3/7 reagent was added simultaneously (Essen Bioscience). Treatments were done in triplicate. Incubation was done in a humid incubator at 37°C and 5% CO_2_ with the IncuCyte live cell imaging system (Essen Bioscience), which allowed us to capture one image from each well through a ×20 objective and GFP filter cube at 2-hour time intervals for 48 hours. The first and last images of each image set were extracted for analysis with Definiens Developer version 1.5 (Definiens Inc., Munich, Germany), and caspase 3/7-positive cells were identified and segmented with an autothreshold segmentation algorithm. This segmentation was further refined by object size, and finally the number of caspase 3/7 cells was enumerated. Values are represented as the average number of caspase 3/7-positive cells from three independent experiments.

### Cell cycle analysis

A549 cells were seeded at 3×10^5^ cells per well in 6-well culture plates. After 24 hours, cells were treated with 5 ng/mL TGFβ-1, 100 nM dasatinib, or vehicle (DMSO). After treatment, floating cells were collected and later combined with adherent cells harvested by trypsinization. Cells were resuspended in 1X PBS, fixed in 2 mL of ice-cold 70% ethanol, and incubated for 2 hours at −20°C. Cell pellets were collected by centrifugation (5,000 rpm for 5 minutes) and resuspended in 400 µL of propidium iodide (0.6 mM), Triton X-100 (0.1% v/v), and RNAse A (0.2 mg/mL). After a 30-minute incubation at 37°C, the cells were analyzed for DNA content using a FACS Calibur (BD Bioscience, San Jose, CA), and data were analyzed using ModFit LT V3.3.11 software (Verity Software House, Topsham, ME).

### SiRNA experiments

ON TARGETplus SMART pool siRNA against *Shc1*, *Smad3*, and negative control were purchased from Dharmacon (Thermo Scientific). Each pool was reconstituted in 1X siRNA buffer (Dharmacon) and diluted in DEPC-treated water to a final concentration of 20 µM. Transient transfections were performed using Lipofectamine RNAiMax (Invitrogen, Grand Island, NY). Briefly, cells were seeded in 6-well plates and transfected after 24 hours at 60–70% confluency. The lipofectamine RNAiMAX, OptiMEM (Invitrogen), and 200 pmol of siRNA mixture were incubated for 20 minutes at room temperature and added to the cells incubated in serum-free media. After incubation at 37°C for 4 hours, the medium was removed. Cells were then washed once in 1X PBS, and compound containing media was added to the cells. Cells were harvested 48 hours post-transfection for protein extraction preparation.

## Results

### TβR-I docking studies

**Figure 1:**
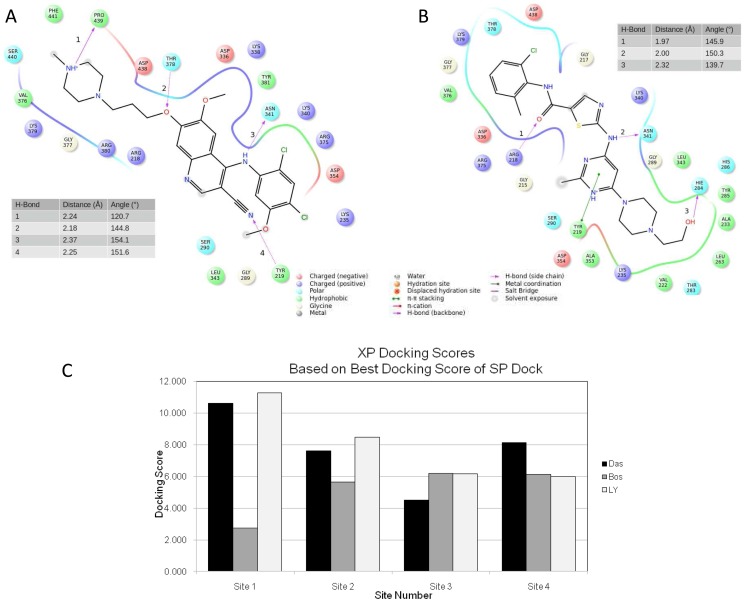
Interactions of dasatinib and bosutinib with TβR-I. Ligand interaction diagram of (A) bosutinib and (B) dasatinib docked into TβR-1. Ligand is represented in black. Hydrogen bonds are labeled by numbers next to the bond. Distances and angles of each hydrogen bond as labeled in the diagram are given in tables within each figure. (C) Site 1 IFD results. Average (black) and best (white) IFD scores of the four compounds docked into site 1 based on ∼50 reported poses for each compound. IFDScore is multiplied by -1 for clarity of presentation.

Experiments using drug affinity chromatography to identify molecules that interact directly with dasatinib have identified over 40 kinases, including tyrosine kinases, receptor tyrosine kinases, serine/threonine kinases, and MAP kinases. One of the serine/threonine kinases identified using this approach is TβR-I [17]. TβR-I has a cysteine-rich N-terminal domain involved in ligand binding, a single transmembrane helix, a regulatory cytoplasmic juxtamembrane region, and a C-terminal serine threonine kinase domain [36]. To examine the binding of dasatinib to TβR-I, we performed docking studies using the previously reported crystal structure of the TβR-I cytoplasmic domain [18]. As described in Materials and Methods, the site designated as Site 1 was chosen as the more likely binding site. In our studies, we compared the binding of dasatinib, bosutinib (structurally related to dasatinib), LY-364947 (TβR-I kinase commercially available inhibitor [37]), and dorsomorphin (originally used in the TβR-I crystallization studies). Each ligand was docked into Site 1 defined by the ligand's best scoring pose from the initial standard precision docking into Site 1. For each ligand, Induced Fit Docking (IFD) was scored, with multiple (∼50) protein-ligand conformations reported from which the average IFD score was calculated (Fig. 1C). Our docking results of the four ligands of interest showed that dasatinib scored the highest. IFD protocol reported that TβR-I -dasatinib formed the best scoring complex (IFD score of approximately -14,742 kcal/mol) as well as scored better on average (IFD score of approximately -14,694 kcal/mol) over all reported complexes. The best TβR-I-dorsomorphin complex had an IFD score of about −14,519 kcal/mol with the average of −14,469 kcal/mol. Bosutinib and LY-364947 performed the poorest, with best protein-ligand complexes scoring at approximately -14,353 kcal/mol and −14,154 kcal/mol, respectively, and averaging −14,313 kcal/mol and −14,119 kcal/mol. Analysis of the top pose produced by the flexible docking model reveals that bosutinib's interaction with the binding site involves four hydrogen bonds with residues Pro-439, Thr-378, Asn-341 and Tyr-219 at distances of 2.4, 2.18, 2.37 and 2.25 Å and with respective angles of 120.7, 144.8, 154.1 and 151.6 degrees (Fig. 1A). Conversely, dasatinib forms three hydrogen bonds with Arg-218, Asn-341 and His-284 at distances of 1.97, 2.00 and 2.32 Å and angles of 145.9, 150.3 and 139.7 degrees (Fig. 1B).

### Dasatinib affects the response to TGFβ-1 in NSCLC cell lines

**Figure 2:**
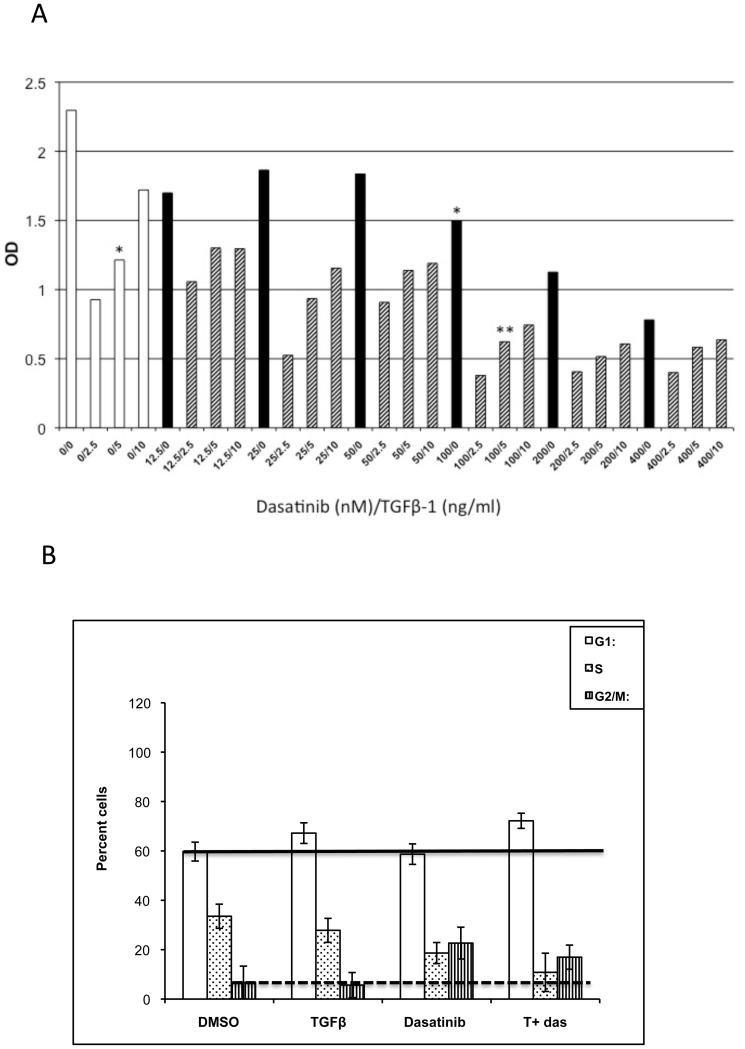
Inhibition of cell growth of A549 cells treated with TGFβ-1 and dasatinib. (A) A549 cells were treated with DMSO, different concentrations of TGFβ-1 (0–10 ng/mL; white bars), different concentrations of dasatinib (0–400 nM; black bars), or a combination of TGFβ-1 and dasatinib (dashed bars) for 48 hours. Treatment of cells with both agents resulted in increase inhibition (**), as compared to when cells were treated with either agent alone (*). (B) A549 cells were treated with DMSO, 5 ng/mL TGFβ-1, 100 nM dasatinib, or a combination of 5 ng/mL TGFβ-1 and 100 nM dasatinib for 48 hours. After 48 hours, propidium iodide stained cells were analyzed to determine cell cycle distribution and analyzed as described in Materials and Methods.

To determine the potential biological relevance of the association between dasatinib and TβR-I, A549 lung cancer cells grown in complete media containing TGFβ-1 were incubated in the presence or absence of different concentrations of dasatinib as described before[9]. Single treatment with TGFβ-1 or dasatinib inhibited cell proliferation (45% and 34%, respectively; Fig. 2A, asterisks). Interestingly, this inhibition of proliferation increased when cells were treated with a combination of TGFβ-1 and dasatinib (75% inhibition; Fig. 2A, double asterisks). The increase in inhibition of proliferation was dependent on the dasatinib dose. Similar results were obtained when cell viability was used to determine number of cells (S1 Figure). To further understand the observed inhibition in cell proliferation, we examined the effect of this combined treatment on cell cycle progression. After 48 hours of treatment with TGFβ-1 and dasatinib, the increase in cells in G1 was not significantly different from when the cells were treated with TGFβ-1 alone (Fig. 2B, solid line). Similarly, with the double treatment, there was an increase in the number of cells in G2/M after TGFβ-1 treatment, but the increase in cells was not different from when cells were treated with dasatinib alone (Fig. 2B, dashed line). Therefore, the reduction in cell number after the TGFβ-dasatinib combination treatment cannot be explained by changes in cell cycle.

### Increased apoptosis in cells treated with TGFβ in combination with dasatinib

**Figure 3:**
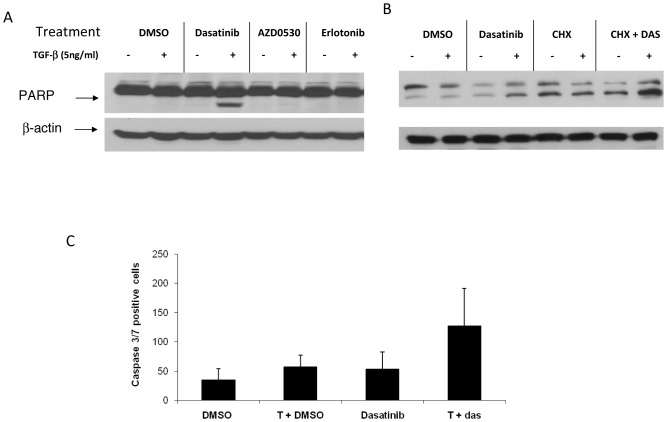
Induction of apoptosis after treatment with TGFβ-1 and dasatinib. (A) A549 cells were treated with 100 nM of dasatinib, 1000 nM of AZD0530, or erlotinib with or without 5 ng/mL TGFβ-1 for 48 hours. (B) A549 cells were pre-treated with 10 µg/mL cycloheximide (CHX) for 1 hour, followed by TGFβ-1 plus or minus dasatinib. After incubation, cells were harvested, lysed, and PARP cleavage detected by Western Blot analysis. (C) A549 cells were seeded in 96-well plates at 5×10^3^ per well. *C*ells were treated, and Cell Player 96-Well Kinetic Caspase 3/7 Reagent was added simultaneously. Treatments were done in triplicate. Values are shown as the average number of caspase 3/7 positive cells from 3 independent experiments.

TGFβ has been implicated in pro-apoptotic responses, as well as anti-apoptotic responses [38]; therefore, we examined whether the inhibition of proliferation seen after combined TGFβ + dasatinib in A549 cells was the result of apoptotic cell death. Apoptosis was measured by poly(ADP-ribose) polymerase (PARP) cleavage after treatment with the TGFβ-dasatinib combination. Dasatinib or TGFβ-1 alone did not induce apoptosis as measured by PARP cleavage (Fig. 3A). This is in agreement with what has been reported before, where treatment of A549 cells with dasatinib did not result in apoptosis [4]. In contrast, the combination of TGFβ + dasatinib resulted in apoptosis, specific to co-treatment with dasatinib, as incubation with an EGFR inhibitor (erlotinib) or another SRC inhibitor (AZD0530) did not result in PARP cleavage (Fig. 3A). To assess whether *de novo* protein synthesis is required for TGFβ + dasatinib-induced apoptosis, A549 cells were pre-treated with 10 µg/mL cycloheximide for 1 hour, followed by TGFβ-1 treatment with or without dasatinib. As shown in Fig. 3B, addition of cycloheximide did not inhibit the increase in apoptosis, suggesting that *de novo* protein synthesis is not required. We examined the effect of the combination treatment in 15 NSCLC cell lines (13 EGFR WT and 2 EGFR MU) and found increased PARP cleavage in 5 of them when treated with the combination TGFβ + dasatinib treatment (S2 Figure).

To confirm the TGFβ + dasatinib-induced apoptosis, a caspase-3/7 assay (Incucyte) was used to simultaneously measure cell death and caspase 3/7 activity. As shown in Fig. 3C, the TGFβ + dasatinib combination treatment resulted in a significant increase (92%) in the number of cells undergoing apoptosis when compared to either treatment alone (22% and 19%, respectively). In addition, the TGFβ-dasatinib combination resulted in loss of mitochondrial electric potential and release of cytochrome c from the mitochondria (data not shown), suggesting that the apoptosis observed is the result of activation of intrinsic (mitochondrial) apoptotic pathways.

### TGFβ-1-dasatinib effect on the activity of TGFβ intracellular mediators

**Figure 4:**
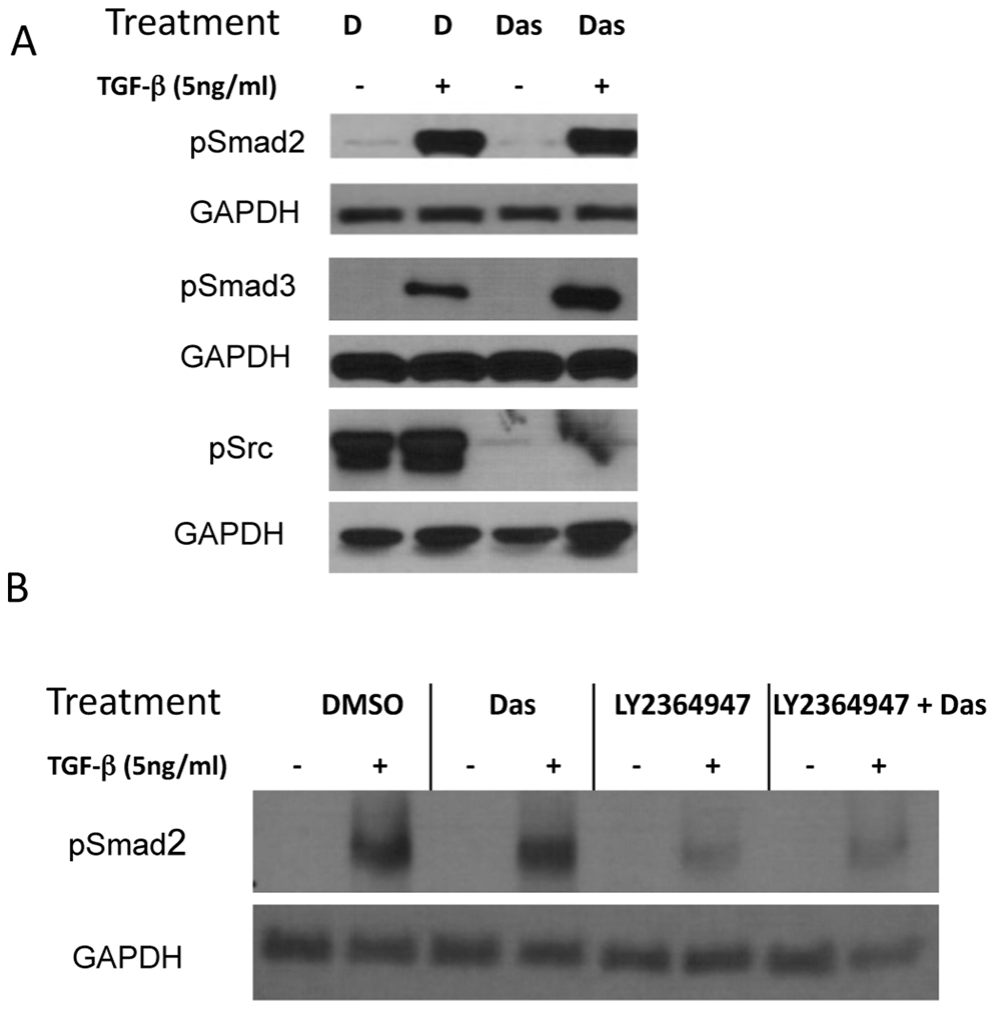
Combination TGFβ-1 and dasatinib treatment effect on phosphorylation of canonical and non-canonical TGFβ pathway intermediaries. A549 NSCLC cells were treated with DMSO, 5 ng/mL TGFβ-1, 100 nM dasatinib, or a combination of 5 ng/mL TGFβ-1 and 100 nM dasatinib for different amounts of time (1 hour for detection of pSmad2 and pSmad3, and 48 hours for detection of pSrc). After incubation, whole cell lysates were collected and subjected to Western blotting with the indicated antibodies.

TGFβ has been shown to stimulate several intracellular pathways, including the Smad pathway (canonical pathway) and other intracellular mediators, including p38 mitogen-activated protein kinase (MAPK), AKT, and ERK (non-canonical pathways)[39]. Therefore, we next examined whether TGFβ-dasatinib treatment activated these pathways by using antibodies that detect the phosphorylated (activated) form of the mediators of both the canonical and non-canonical pathways. As shown in Fig. 4, expression of activated Smad2 or Smad3 was not observed in whole cell extracts of either untreated or dasatinib-treated cells but was observed after TGFβ-1 treatment or after treatment with TGFβ + dasatinib. Interestingly, levels of phosphorylated Smad3 after treatment with TGFβ-dasatinib are higher than levels seen with TGFβ-1 treatment alone. The increase in p-Smad3 seen after 1 hour of TGFβ-dasatinib combination treatment in Smad3 activation is transient as it cannot be seen 48 hours after treatment (data not shown). This is in contrast to Smad2 phosphorylation, which can be seen as early as 5 minutes and remains phosphorylated until 48 hours. This could be due to the considerably shorter half-life of Smad3 (4.7 hours) compared to Smad2 (12.5 hours)[40]. Pre-treatment with the TβR-I inhibitor LY364947, blocks Smad2 phosphorylation in the presence or absence of dasatinib (Fig. 4B). As expected, SRC phosphorylation is inhibited in the presence of dasatinib, and incubation with TGFβ-1 does not affect this inhibition.

**Figure 5:**
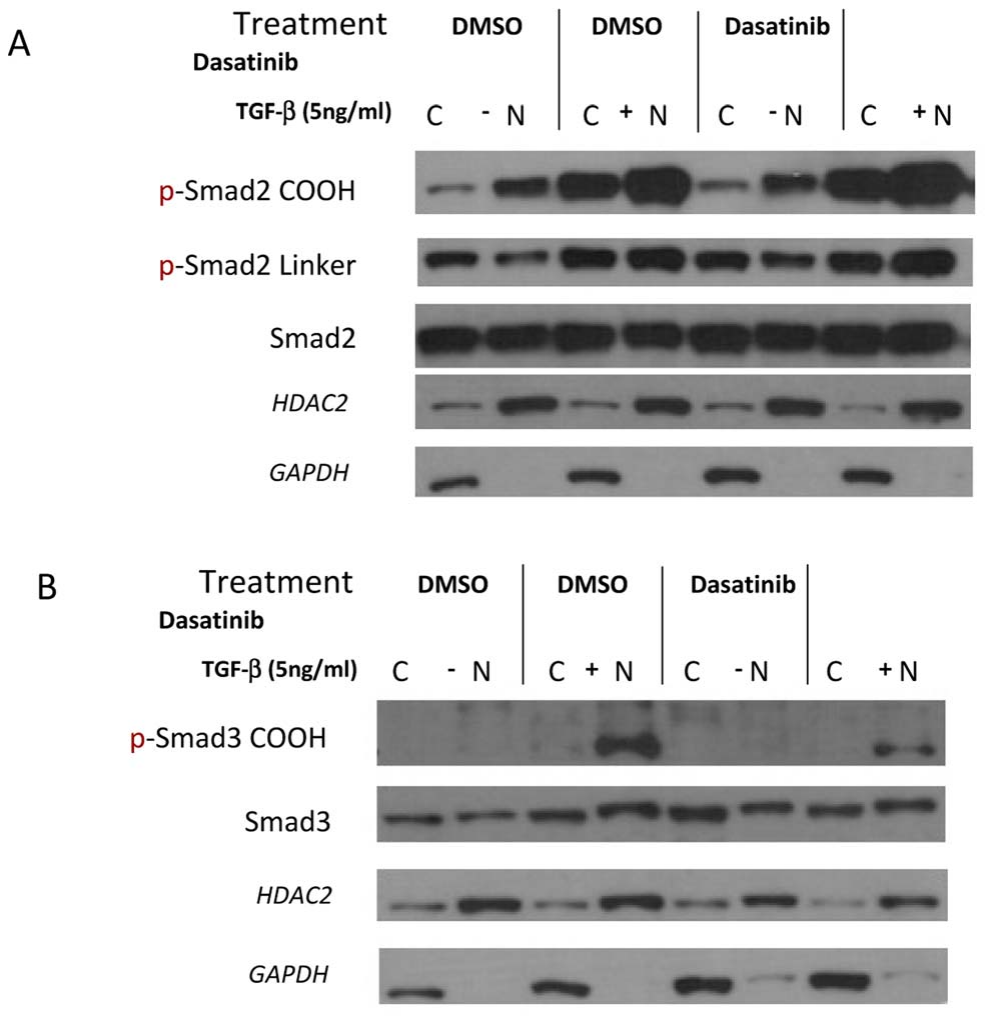
Dasatinib does not affect the intracellular localization of phosphorylated Smads phosphorylation of non-canonical intermediaries after TGFβ treatment. A549 cells were treated with DMSO, 5 ng/mL TGFβ-1, 100 nM dasatinib, or a combination of 5 ng/mL TGFβ-1 and 100 nM dasatinib for 48 hours. After treatment, cell lysates were collected and the nuclear and cytoplasmic fractions were separated as described before [35]. After fractionation, lysates were subjected to Western blotting with the indicated antibodies.

Localization and activity of Smads proteins are dependent of the phosphorylation status in the COOH and linker regions [41]. To determine whether the increase in apoptosis observed with the combination treatment is due to alterations in the location of the Smads, we examined the effect of the combined TGFβ-dasatinib treatment on localization of the active Smads. After 48 hours of TGFβ-1 or combination treatment of TGFβ-dasatinib, cell extracts were fractionated and the phosphorylated Smads were examined in each fraction. As shown in Fig. 5A, the Smad2 phosphorylated in the carboxy termini (p-Smad2 COOH) increases predominantly in the nucleus. The Smad2 phosphorylated in the linker region (p-Smad2 Linker) also increases after treatment with TGFβ-1, but a greater amount remains in the cytoplasm. Interestingly, the amount of phosphorylated Smad3 after TGFβ-1 treatment also accumulates in the nucleus, independent of dasatinib treatment (Fig. 5B).

Treatment with combination TGFβ-dasatinib did not have a significant effect on the non-canonical TGFβ pathway intracellular mediators ERK, AKT, or p38 (S3 Figure). In addition, treatment with TGFβ-1 did not result in an increase in Shc activation in A549 cells, as previously reported for other cell types [42], [43], and knockdown of the expression of ShcA using ShcA siRNA did not have an effect in apoptosis in ShcA-negative A549 cells (data not shown), suggesting that this pathway is not involved in the induced apoptosis observed with TGFβ-dasatinib treatment.

### TGFβ-1-dasatinib apoptosis is mediated by Smad3-induced BIM

**Figure 6:**
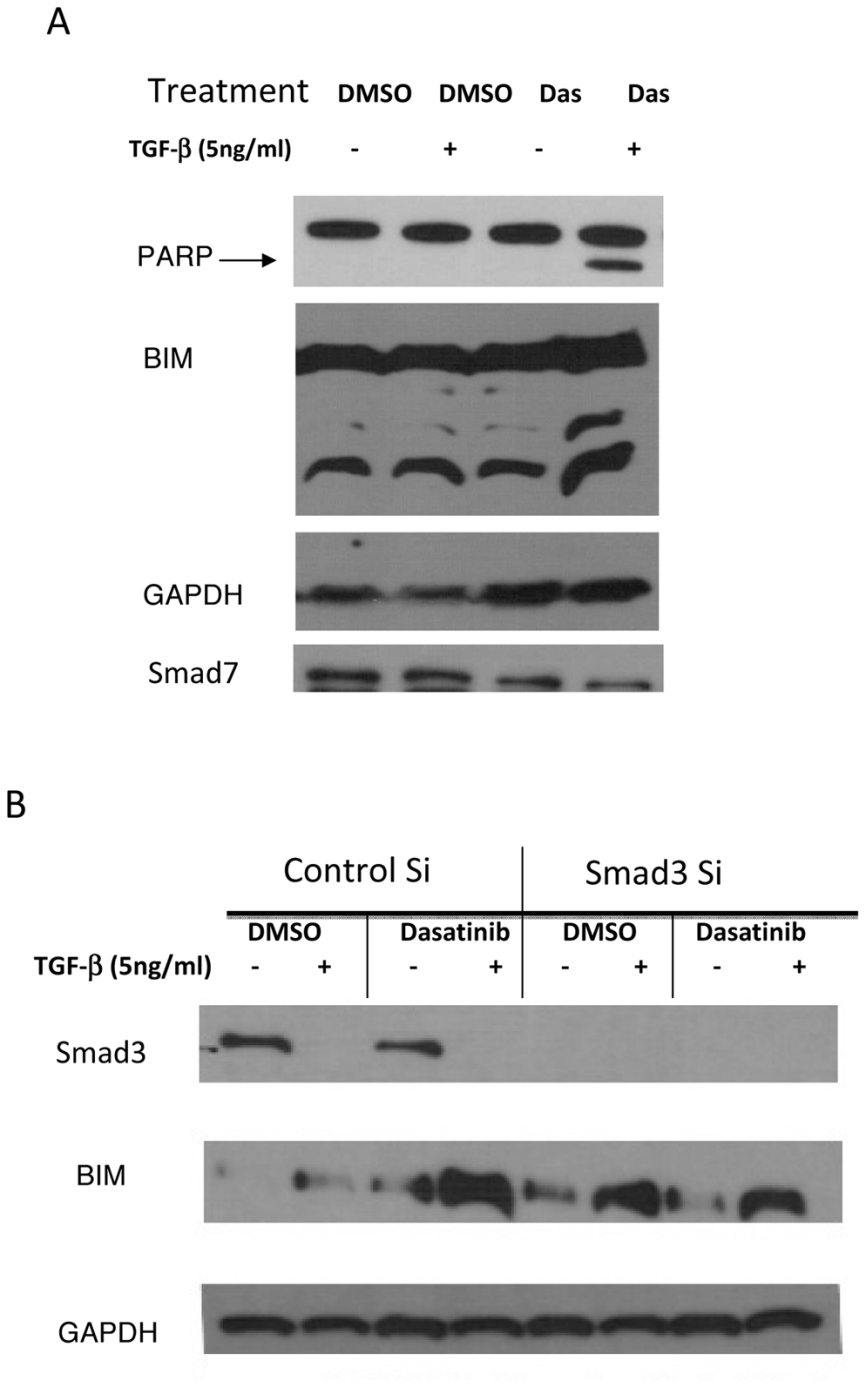
Increase in BIM after combination treatment mediated by Smad3. (A) A549 cells were treated with DMSO, 5 ng/mL TGFβ-1, 100 nM dasatinib, or a combination of 5 ng/mL TGFβ-1 and 100 nM dasatinib for 48 hours. After treatment, whole cell lysates were collected and subjected to Western blotting with the indicated antibodies. (B) siRNA against Smad3, and negative control were transfected into A549 cells. After 4-hour incubation, cells were washed and media containing compounds were added to each well. Cells were harvested 48 hours post-transfection for protein extraction preparation and Western blotting analysis. Figure is representative of 3 independent experiments.

Smad3 has been reported to sensitize cells to the apoptotic effects of TGFβ [40], and one of the suggested mechanisms is the induction of the expression of the pro-apoptotic protein BIM (Bcl-2- interacting mediator of cell death)[44]. As shown in Fig. 6A, at 24 hours after combination treatment, we observed an increase in BIM protein expression of the high molecular weight isoform and of the lower molecular and more cytotoxic BIM. It has recently been reported that a BIM deletion polymorphisms (resulting in a BIM isoform without the BH3 domain) are involved in resistance to tyrosine kinase inhibitors in several cancer types [45], [46]. Screening of all 15 cell lines using primers described in the literature [45] we were not able to detect the BIM isoform in any of our cell lines (S5 Figure). However, knockdown of the expression of Smad3 using Smad3 siRNA resulted in a decrease in the amount of BIM after combination treatment, indicating that Smad3 expression is necessary for increased expression of BIM (Fig. 6B). It has been reported that expression of Smad7 prevents apoptosis by inhibiting the expression of BIM [47]. As can be seen in Fig. 6A with the TGFβ-dasatinib treatment that resulted in PARP cleavage, we observed reduced expression of Smad7. This decrease in Smad7 suggests a mechanism to keep the TGFβ pathway active. Our data suggest that the combination treatment of TGFβ and dasatinib induces apoptosis by increasing TGFβ activation of BIM and down-regulation of TGFβ pathway inhibitory signals, such as Smad7.

## Discussion

Dasatinib (BMS-354825, Sprycel), a SRC TKI, is currently used in the treatment of chronic myeloid leukemia and is in clinical trials for osteosarcoma, NSCLC, and other cancers. At low concentrations (IC_50_<1.0 nM), dasatinib inhibits Abl and SRC family kinases, and at higher concentrations it inhibits other tyrosine kinases, including p38 MAPK, AKT, FAK, PDGFR, and Ephrin[48]. In NSCLC, dasatinib is only effective in killing EGFR MU cells [9], which account for 10% of NSCLCs. Experiments using drug affinity chromatography to identify molecules that interact directly with dasatinib have identified TβR-I [17]. Here, we show that dasatinib is likely to bind in the same pocket as the TGFβ receptor inhibitor LY-364947, but it does not inhibit the signaling of TGFβ. Interestingly, combination treatment with dasatinib and TGFβ results in an increase in TGFβ-induced apoptosis, mediated by the pro-apoptotic BH3-only protein, BIM.

Several approaches have been developed for blocking TGFβ signaling and drugs have been developed that are either in nonclinical or in early stages of clinical investigation [49]. Strategies for inhibition of the TGFβ signaling include compounds that interfere with the binding of TGFβ to its receptors, drugs that block intracellular signaling, and antisense oligonucleotides. Strategies that block catalytic activity of TβR-I, including small molecules such as SB-431542 and SB-505124 (GlaxoSmithKline), SD-093 and SD-208 (Scios), and LY-580276 (Lilly Research Laboratories), act as competitive inhibitors for the ATP-binding site of the TβR-I kinase [50], [51]. In addition, there are a series of compounds originally identified as SRC inhibitors that directly interact with TβR-I. The SRC kinase inhibitors PPI and PP2 have been reported to block TGFβ-induced Smad phosphorylation as well as TGFβ down-regulation of e-cadherin by inhibiting the kinase activity of TβR-I [52]. In contrast to specific SRC inhibitors such as SU6656 and AZD, dasatinib is more similar to PPI and PP2, which have other targets in addition to SRC. However, although many of these inhibitors look promising in pre-clinical studies, the dual role of TGFβ in tumorigenesis requires an understanding of the interactions of the TGFβ pathway with other pathways in order to design successful therapeutic approaches without undesired side effects.

**Figure 7:**
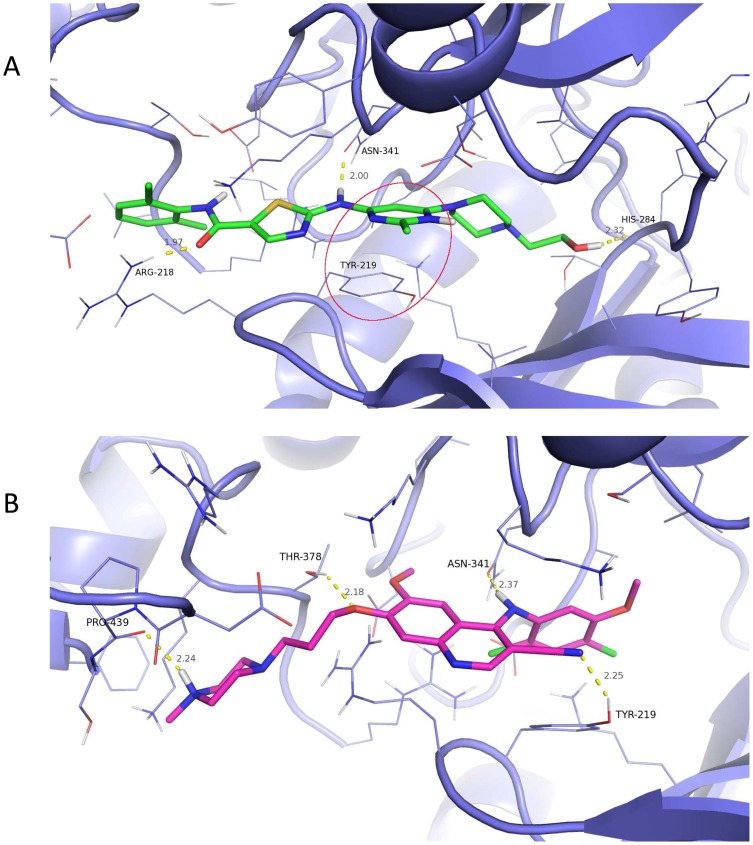
3D Docked Pose and Interactions of dasatinib and bosutinib with TβR-I 3D rendering of the binding pose of (A) dasatinib and (B) bosutinib docked into TβR-1. Protein is represented by purple carbon cartoon representation with residues surrounding a ligand are in line representation. Bosutinib is colored with magenta carbons and dasatinib with green carbons. Hydrogen bonds represented by dashed yellow lines with distances (gray) and interacting residue (black) labeled. A π-π stacking interaction between the phenyl of TYR-219 and the pyrimidine of dasatinib is circled in red.

Based on our docking studies we hypothesize that, dasatinib is a stronger binder to TβR-I when docked into the site characterized by the dorsomorphin co-crystallized with the kinase domain of TβR-I. This pocket, according to our modeling, is a likely binding site for the four ligands computationally probed against TβR-I. Our hypothesis, supported by the previous results of ligand affinity chromatography, and stronger binding of dasatinib versus bosutinib, is in agreement with the binding experiments. Based on the structural analysis of the docking results, stronger binding of dasatinib relative to bosutinib may largely contribute to an extra π-π stacking interaction between the phenyl of TYR-219 and pyrimidine of dasatinib. The 3D pose view of dasatinib and bosutinib is presented in Figs. 7A and 7B respectively. Although docking of bosutinib reveals an extra hydrogen-bond, overall the intra-molecular hydrogen bonds formed in the complex are farther apart and some with less favorable geometry which diminishes their contribution to binding when compared to hydrogen bonding between dasatinib and TβR-I.

It has been reported that treatment of NSCLC lines with dasatinib results in apoptosis; however, apoptosis was reported in NSCLC cell lines with a MU EGFR phenotype, but not in cell lines with a WT EGFR phenotype [9]. Because the A549 NSCLC cell line used in our studies has a WT EGFR phenotype, we speculated whether the apoptotic cell death resulting from the combination treatment might be a novel response of NSCLC cells that exhibit a WT EGFR phenotype. However, this effect was not exclusive to EGFR WT cell lines, as several other lung cancer cell lines with a WT EGFR phenotype did not show PARP cleavage specific to TGFβ-dasatinib treatment. Interestingly, the majority of the cell lines that cleaved PARP as response to the combination treatment were those capable of going through TGFβ-induced epithelial-to-mesenchymal transition (EMT). PARP cleavage specific to the TGFβ-dasatinib combination was observed in three out of four cell lines tested that undergo EMT (A549, H1944 and PC-9 cells) but not in cell lines that do not undergo EMT (e.g., H292, H1935) (S2 Figure). It is not surprising that response to a TKI inhibitor correlates with EMT capacity, as there are several reports in the literature that demonstrate that EMT plays a role in acquired resistance to TKIs. For example, H4006 cells resistant to erlotinib have been shown to acquire a mesenchymal phenotype and down regulate e-Cadherin. In addition, dasatinib has been reported to inhibit migration of melanoma [53] and sarcoma [54] cell lines, and treatment of A549 cells with dasatinib resulted in inhibition of TGFβ-1-induced migration. In addition, dasatinib has been reported to block migration and invasion of the breast cancer cell lines MDA-MD-231 when administered in combination with doxorubicin [3]. Interestingly, treatment of A549 cells with the TGFβ-dasatinib combination has no effect on the regulation of e-cadherin by TGFβ-1 (data not shown). This is also true for the H292 NSCLC cell line, which does not undergo EMT, and treatment with TGFβ-1 does not result in changes in the expression of e-cadherin. Co-treatment of H292 cells with TGFβ-1 and dasatinib does not result in changes in e-cadherin expression, signifying that dasatinib co-treatment has no effect on the TGFβ regulation of e-cadherin expression (data not shown). Further experiments to examine the effect of the combination treatment in other molecules and responses involved in TGFβ- induced EMT (e.g. invasion) are currently under investigation.

TGFβ has been shown to induce apoptotic cell death in a variety of cells types through different pathways, including the activation of Smads, BIM, and the caspase cascade [38]. The pro-apoptotic BIM belongs to the BH3-only subgroup of the Bcl-2 family, and it can be regulated at both the transcriptional and post-transcriptional levels. At the transcriptional level, BIM is regulated by serum, growth factors, and cytokines. In gastric cells, knockdown of Smad3 suppresses expression of BIM, supporting a role for Smad3 in the transcriptional regulation of BIM [55]. Our data show that NSCLC treatment with a combination of TGFβ and dasatinib results in up-regulation of BIM in cell lines where there is increase PARP cleavage (A549, PC-9 and H1944). This has been previously reported in lymphoid cells, where at the same time that TGFβ induces BIM transcriptionally, it attenuates ERK phosphorylation and thus inhibits BIM phosphorylation. However, in our experiments, we did not see an effect of the combined TGFβ-dasatinib treatment on ERK phosphorylation (S3 Figure) but did see a decrease in Shc phosphorylation, which have been described as a key protein in ERK activation.

Smad3 binds DNA in response to TGFβ and regulates transcription, resulting in apoptosis [40], [44]; however, only a transient increase in Smad3 phosphorylation was seen with the combination TGFβ-dasatinib treatment, suggesting that other mechanisms might be involved in the response still seen at 48 hours. Treatment of TGFβ-responsive luciferase reporter constructs with the combination of TGFβ-dasatinib resulted in a decrease in luciferase activity (S4 Figure), suggesting that transcriptional repression of certain genes could be involved in the apoptotic responses seen. This is in agreement with the cyclohexamide experiments (Fig. 3) that suggested that no new protein synthesis is needed for the increase in apoptosis seen after the TGFβ-dasatinib combination treatment. Decreased expression of a negative regulator of the TGFβ pathway (e.g., Smad7) could be one of the mechanisms involved in the decrease levels of BIM seen after TGFβ-dasatinib treatment. Interestingly, while it has been reported that expression of Smad7 is needed for TGFβ-mediated apoptosis in normal epithelial cells (HaCaT) and prostatic carcinoma cells (PC-3U), in human tissues Smad7 activity is correlated to tissue specificity. It was recently reported that the levels of expression of Smad7 can be prognostic in human tumors in a tissue-dependent manner. In breast and melanoma tumors, increased levels of Smad7 are associated with a better outcome [56], [57], while in colon and hepatic cancer, increased levels of Smad7 are associated with worse outcome [58], [59].

Combination TGFβ-dasatinib treatment affects the canonical pathway protein (Smad3, Smad7) and non-canonical pathway (Shc). Therefore, it might reflect not an overlap or dependence on any of the individual pathways (because we did not see apoptosis with either TGFβ alone or with dasatinib alone) but both pathways working simultaneously to result in apoptosis, as reported before [60]. Identification of other molecules (and their phosphorylation status) that are differentially expressed when dasatinib treated cells are also treated with TGFβ will provide us with clues to identify other pathways involved in the induction of apoptosis. Differential expression and phosphorylation states could in turn be used as biomarkers to determine response to the combination treatment in a clinical setting. These type of experiments will help understand the molecules and mechanisms involved in the pleiotropic TGFβ responses, as well as devise novel combination treatments for lung cancer.

Dasatinib has shown minimal activity as a single agent in patients with advanced lung cancer. Our results in lung cancer cell lines lacking activating EGFR mutations are consistent with these clinical findings, as cells do not undergo apoptosis but rather have growth arrest. Thus, to maximize the effects of Src inhibition in lung cancer, combination therapy approaches would be necessary. Our results suggest that some cells that have undergone EMT may have heightened sensitivity to dasatinib but the effect is still cytostatic. Further experiments could delineate other key pathways activated in these EMT lines to propose rationale combination therapies with Src inhibitors. Preclinical experiments are needed before any clinical trials should be proposed.

## Supporting Information

S1 Figure **A549 cells were trypzined, spun and resuspended in 0.5 mL 1X PBS, after which they were stained with Trypan blue.** Live cells were calculated after counting in a hemocytometer. Figure represents number of cells in each treatment.
(TIF)

S2 Figure **(A) H23, H292, H322, H358, H441, H522, H1437, H1395, H1648, H2347, HCC4006, PC9, A549, H1944 and Calu-6 NSCLC cells were treated with 100 nM of dasatinib, with or without 5 ng/mL TGFβ for 48 hours.** After incubation, cells were harvested, lysed, and PARP cleavage detected by Western Blot analysis (arrows). Solid arrows denotes uncleaved PARP and dash arrows denotes cleaved PARP.
(TIF)

S3 Figure **Combination TGFβ-1 and dasatinib treatment effect on phosphorylation of non-canonical TGFβ pathway intermediaries.** A549 NSCLC cells were treated with DMSO, 5 ng/mL TGFβ-1, 100 nM dasatinib, or a combination of 5 ng/mL TGFβ-1 and 100 nM dasatinib for different amounts of time (3 hours for detection, pERK and p38; 48 hours for detection of pAKT and pERK). After incubation, whole cell lysates were collected and subjected to Western blotting with the indicated antibodies.
(TIF)

S4 Figure **Combination TGFβ-1 and dasatinib treatment reduces TGFβ transcriptional responses.** TGFβ-induced transcription is inhibited by co-treatment with TGFβ in transient and transfections with TGFβ-responsive luciferase constructs. A549 cells were transiently transfected with p3TP-Lux reporter or pSBE4 and treated with DMSO, 5 ng/mL TGFβ-1, 100 nM dasatinib, or a combination of 5 ng/mL TGFβ-1 and 100 nM dasatinib for 48 hours.
(TIF)

S5 Figure **PCR detection of** ***BIM*** **polymorphic deletion.** Genomic DNA was obtained form the NSCLC used in this study and amplified with two different sets of primers that distinguish the wild-type and deletion BIM polymorphism alleles. As can be seen only the wild-type allele was amplified in all of the cell lines included in our studies. HCC2279 cell line, which has been reported to express the polymorphic allele (Ng *et al*, 2012; reference # 45 in manuscript), was the only cell line in which the polymorphic allele was amplified.
(TIF)

S1 Materials and Methods **Materials and methods for S1-S5 Figures.**
(DOCX)
