# Supplementary figures and images for: Transforming Growth Factor β Signaling Overcomes Dasatinib Resistance in Lung Cancer
Source: PLoS One. 2014 Dec 11;9(12):e114131. doi: 10.1371/journal.pone.0114131 (PMC4263601; doi:10.1371/journal.pone.0114131)

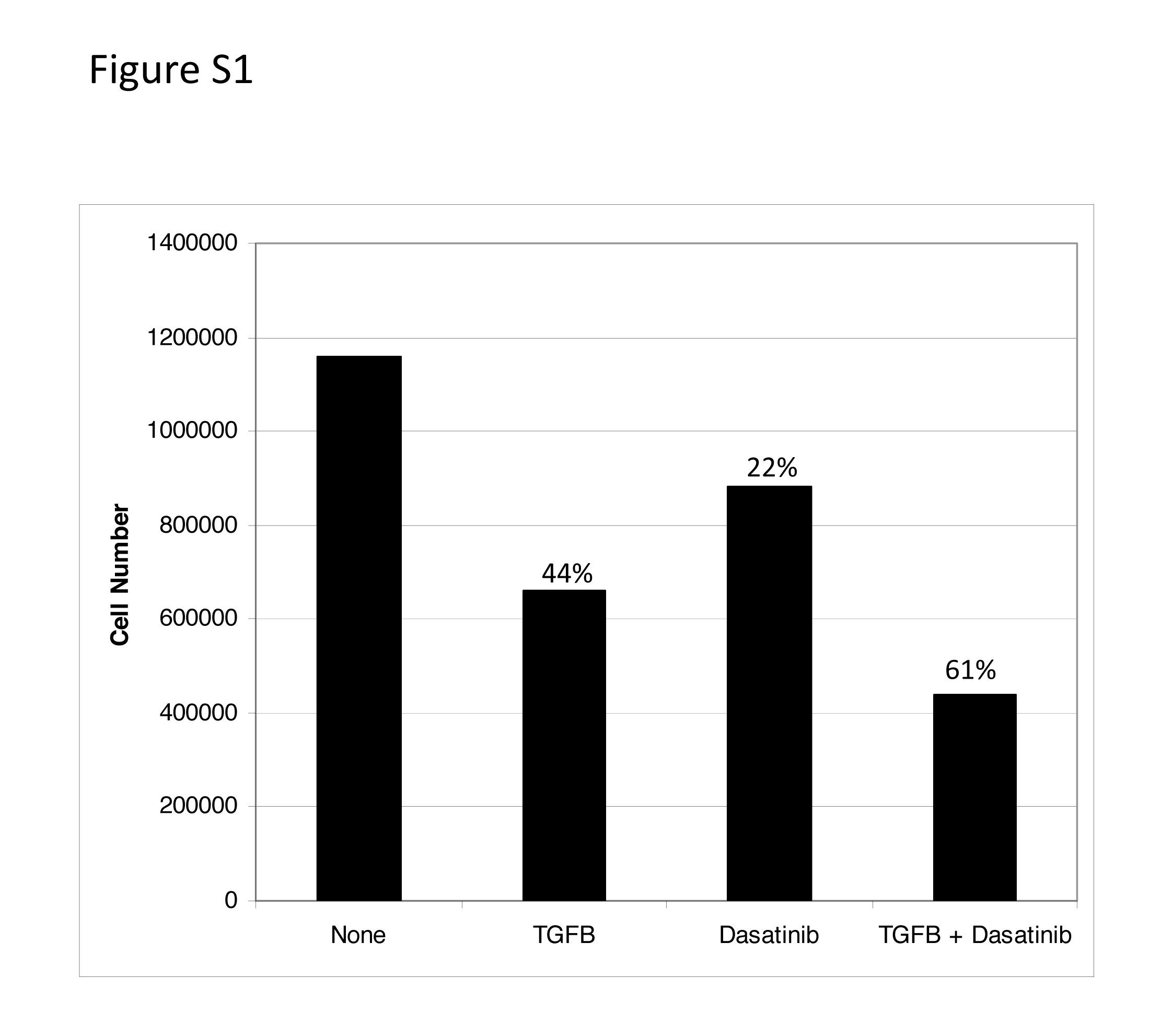

Supplement: S1 Figure — A549 cells were trypzined, spun and resuspended in 0.5 mL 1X PBS, after which they were stained with Trypan blue. Live cells were calculated after counting in a hemocytometer. Figure represents number of cells in each treatment. (TIF) [file pone.0114131.s001.tif]

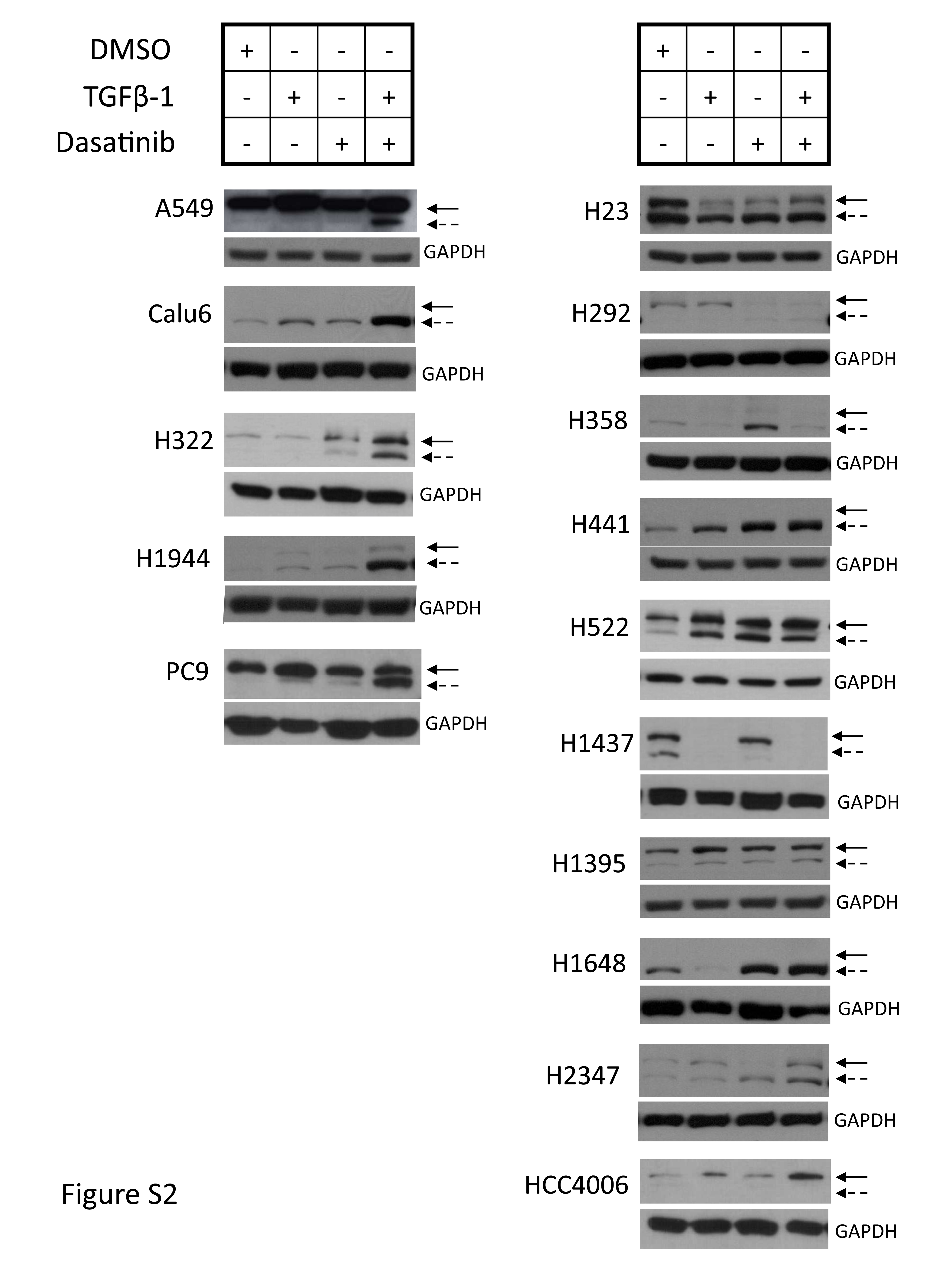

Supplement: S2 Figure — (A) H23, H292, H322, H358, H441, H522, H1437, H1395, H1648, H2347, HCC4006, PC9, A549, H1944 and Calu-6 NSCLC cells were treated with 100 nM of dasatinib, with or without 5 ng/mL TGFβ for 48 hours. After incubation, cells were harvested, lysed, and PARP cleavage detected by Western Blot analysis (arrows). Solid arrows denotes uncleaved PARP and dash arrows denotes cleaved PARP. (TIF) [file pone.0114131.s002.tif]

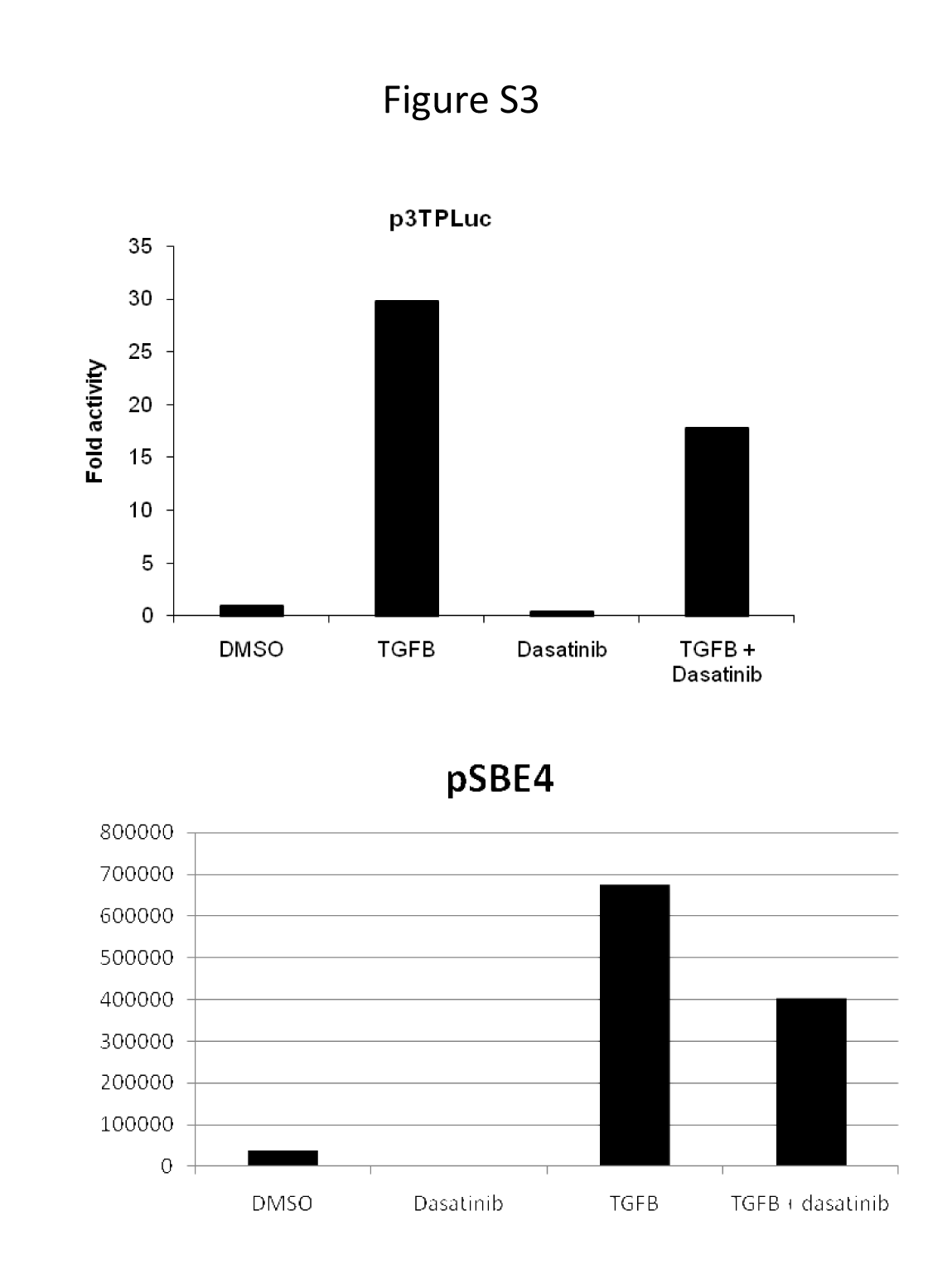

Supplement: S3 Figure — Combination TGFβ-1 and dasatinib treatment effect on phosphorylation of non-canonical TGFβ pathway intermediaries. A549 NSCLC cells were treated with DMSO, 5 ng/mL TGFβ-1, 100 nM dasatinib, or a combination of 5 ng/mL TGFβ-1 and 100 nM dasatinib for different amounts of time (3 hours for detection, pERK and p38; 48 hours for detection of pAKT and pERK). After incubation, whole cell lysates were collected and subjected to Western blotting with the indicated antibodies. (TIF) [file pone.0114131.s003.tif]

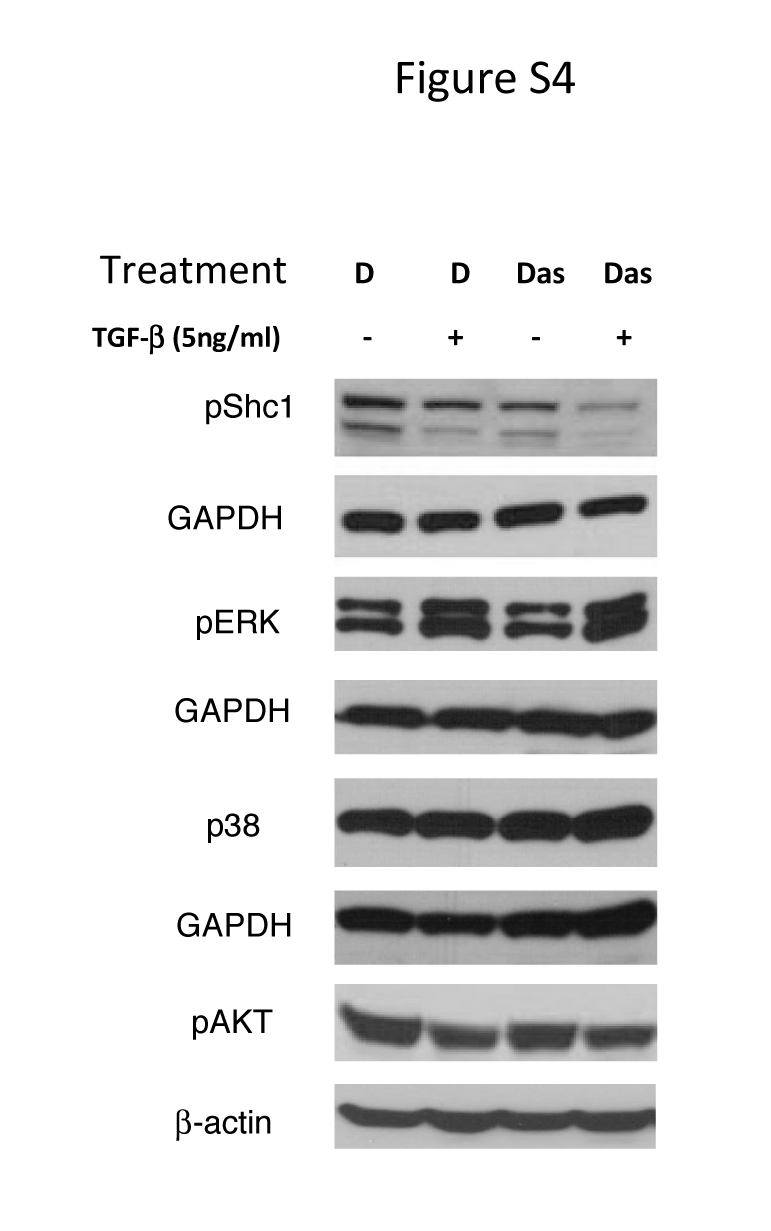

Supplement: S4 Figure — Combination TGFβ-1 and dasatinib treatment reduces TGFβ transcriptional responses. TGFβ-induced transcription is inhibited by co-treatment with TGFβ in transient and transfections with TGFβ-responsive luciferase constructs. A549 cells were transiently transfected with p3TP-Lux reporter or pSBE4 and treated with DMSO, 5 ng/mL TGFβ-1, 100 nM dasatinib, or a combination of 5 ng/mL TGFβ-1 and 100 nM dasatinib for 48 hours. (TIF) [file pone.0114131.s004.tif]

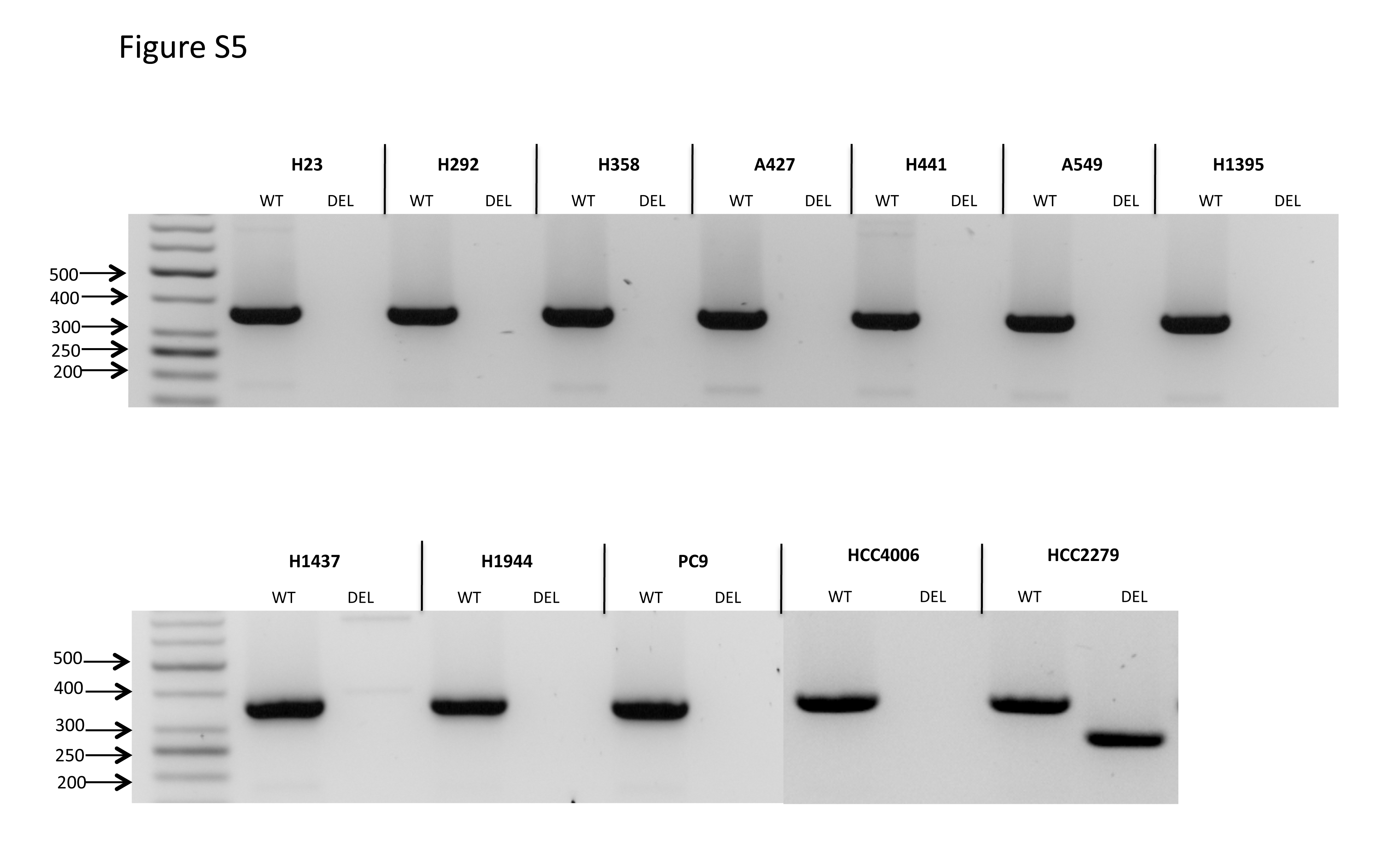

Supplement: S5 Figure — PCR detection of BIM polymorphic deletion. Genomic DNA was obtained form the NSCLC used in this study and amplified with two different sets of primers that distinguish the wild-type and deletion BIM polymorphism alleles. As can be seen only the wild-type allele was amplified in all of the cell lines included in our studies. HCC2279 cell line, which has been reported to express the polymorphic allele (Ng et al, 2012; reference # 45 in manuscript), was the only cell line in which the polymorphic allele was amplified. (TIF) [file pone.0114131.s005.tif]
