# Supplementary material for: Transforming Growth Factor β Signaling Overcomes Dasatinib Resistance in Lung Cancer
Source: PLoS One. 2014 Dec 11;9(12):e114131. doi: 10.1371/journal.pone.0114131 (PMC4263601; doi:10.1371/journal.pone.0114131)
Supplement: S1 Materials and Methods — Materials and methods for S1-S5 Figures. (DOCX) [file pone.0114131.s006.docx]

**SUPPORTING INFORMATION**

**Materials and methods**

**Cell culture:** The following human lung adenocarcinoma cell lines were obtained from the American Type Culture Collection (Manassas, VA): H23, H292, H322, H358, H441, H522, H1437, H1395, H1648, H2347, HCC4006, HCC2279, PC9, A549, H1944 and Calu-6. NSCLC cell lines were cultured in RPMI-1640 medium (Thermo Scientific, Waltham, MA) supplemented with 10% fetal bovine serum (Atlanta Biologicals, Inc, Lawrenceville, GA), 100 U/mL penicillin, 100 μg/mL streptomycin, and 1 mM glutamine. The cell lines were maintained in a humid incubator at 37°C and 5% CO_2_.

**Antibodies and compounds:** Polyclonal anti-Shc1 antibody was obtained from Thermo Scientific Pierce (Rockford, IL). The following antibodies were purchased from Cell Signaling Technology (Danvers, MA): anti-pAKT, anti-pERK, anti-BIM, anti-PARP, and anti-GAPDH. Recombinant human TGFβ-1 protein was purchased from R&D Systems (Minneapolis, MN) and reconstituted in 4 mM HCL and 1 mg/mL bovine serum albumin solution. Dasatinib was obtained from ChemieTek (Indianapolis, IN) and diluted in DMSO.

**Plasmids and Liposome-Mediated Gene Transfer.** The p3TP-Lux luciferase reporter contains three repeats of the TPA-responsive element (TRE) fused to a portion of the PAI-1 promoter (provided by J. Massagué, Sloan Kettering Cancer Center, New York). pSB4-Luc pSBE4-Luc contains four copies of the Smad-binding element (SBE). Transient transfections were performed as described previously (Muñoz-Antonia, T., Li, X., Reiss, M., Jackson, R., and Antonia, S. (1996) *Cancer Res.* **56,** 4831-5) with some modifications. Briefly, plasmid DNA (1.7 μg of the luciferase reporter plasmid) were mixed with 10 μL of Fugene reagent (Roche Diagnostic) and incubated for 15 minutes at room temperature before addition to semiconfluent cell cultures in 60 mm tissue culture dishes. Four hours after the start of transfection, cells were treated with either 5ng/ml TGFβ-1, 100 nM dasatinib or with both 5ng/ml TGFβ-1 and 100 nM dasatinib. Forty-eight hours after the start of the transfection, the amount of luciferase enzyme activity in cell extracts was determined using the Luciferase Assay System (Promega Corporation). Ten μL of the cell extracts were added to 50 μL of the Luciferase reagent and the amount of light produced was measured using a Barthold Luminometer (Wallac, Inc., Gaithersburg, MD). The amount of protein present in the cell extracts was determined using the Bio-Rad Bradford assay.

**PCR detection of *BIM* polymorphic deletion.** We extracted gDNA from NSCLC cell lines (Wizard Genomic DNA Purification Kit, Promega) and genotyped the deletion by two separate PCR reactions that distinguish the wild-type and deletion alleles (Ng *et al*, 2012; reference # 45 in manuscript). The wild-type allele was amplified with the following primers: forward primer 5′-CCA CCAATGGAAAAGGTTCA-3′ and the reverse primer 5′-CTGTCATTTC TCCCCACCAC-3′. The deletion allele was amplified using the forward primer 5′-CCACCAATGGAAAAGGTTCA-3′ and the reverse primer 5′-GGC ACAGCCTCTATGGAGAA-3′. PCR reactions were performed using Jumpstart REDAccuTaq DNA polymerase (Sigma-Aldrich) with the thermo cycling conditions described before by Ng *et al*. The PCR products for the *BIM* polymorphic deletion (284 bp) and the wild-type (362 bp) alleles were analyzed on a 2% agarose gel. The NSCLC cell line HCC2279 was used as a positive control as it contains both alleles.
